# In vitro assays as a tool to personalize treatment in central nervous system tumors: a systematic literature review

**DOI:** 10.1007/s10238-026-02059-w

**Published:** 2026-02-06

**Authors:** Martina Offi, Mariachiara Buccarelli, Silvia Chiesa, Ciro Mazzarella, Maria Laura Falchetti, Giovanni Maria Ceccarelli, Giuliano Di Monaco, Federico Maria Cocilovo, Martina Taglialatela, Sohum Shetty, Alessandro Olivi, Liverana Lauretti, Roberto Pallini, Lucia Ricci-Vitiani, Quintino Giorgio D’Alessandris

**Affiliations:** 1 Department of Neuroscience, Catholic University School of Medicine, Rome, Italy; 2 Department of Neurosurgery, Fondazione Policlinico Universitario A. Gemelli IRCCS, Rome, Italy; 3 Department of Oncology and Molecular Medicine, Istituto Superiore di Sanità, Rome, Italy; 4 Department of Radiation Oncology, Fondazione Policlinico Universitario A. Gemelli IRCCS, Rome, Italy; 5 Institute of Biochemistry and Cell Biology, National Research Council, Monterotondo, Rome, Italy

**Keywords:** Tailored therapy, Cerebral tumors, Glioblastoma, Glioma stem-like cells, ChemoID

## Abstract

Personalized therapy in neuro-oncology has traditionally relied on molecular profiling. However, clinical benefit has been scarce to date. Recently, in vitro drug sensitivity testing using patient-derived models—such as organoids and cell lines—has emerged as a promising strategy. We systematically reviewed evidence on the efficacy of in vitro drug screening in predicting treatment outcome for brain tumors, including but not limited to glioblastoma. PRISMA guidelines were followed. Fifteen studies were included, comprising 300 patients overall. Cohort studies built the largest group; only one randomized clinical trial was found. In vitro assays, using patient-derived stem cells, standardized assays ad the ChemoID, or tumor-derived organoids, were able to reliably predict treatment outcome. However, the overall quality of evidence was limited. These models may overcome limitations of molecular profiling, especially in glioblastoma, where driver mutations are often lacking and the molecular profile evolves at recurrence. Although initial results are promising, further validation is needed before clinical implementation.

## Introduction

Central nervous system tumors, particularly malignant gliomas such as glioblastoma, IDH-wildtype (GBM), represent an unmeet need in clinical oncology. Despite improvements in surgery and radiotherapy and the introduction of advanced therapies, the prognosis for patients with these aggressive tumors remains dismal [1–3]. This poor prognosis is primarily due to the inherent resistance of GBM cells to conventional treatments such as chemotherapy and radiotherapy, as well as the tumors’ extensive heterogeneity. This heterogeneity not only complicates treatment decisions but also contributes to the tumor’s ability to evade therapeutic interventions, ultimately leading to recurrence and treatment failure [3–5].

In recent years, targeted therapy has emerged as a promising strategy for improving the treatment of GBM. This approach aims to specifically target the molecular drivers of tumor growth, such as mutated genes or dysregulated signaling pathways, in contrast to conventional treatments that indiscriminately affect both cancerous and healthy cells [6, 7]. However, despite significant progress in understanding the molecular biology of gliomas, identifying reliable molecular predictors of treatment response remains a critical challenge. This is particularly true for recurrent GBM, where tumors often exhibit further genetic and phenotypic alterations that make them even more resistant to standard therapies [8, 9].

To address this gap, in vitro cell-based assays have been proposed as a valuable tools to better predict the response of brain tumors to various therapies. These assays leverage on patient-derived tumor models, including glioblastoma stem-like cells (GSCs), which represent a small but highly crucial subpopulation of cells within the tumor. GSCs are known to be particularly resistant to both radiotherapy and temozolomide (TMZ), the standard chemotherapy used in GBM treatment [8]. It is believed that GSCs, due to their stem-like properties, contribute significantly to tumor recurrence after treatment, making them a key target for the development of novel therapeutic strategies. Because of their ability to regenerate and fuel tumor growth, GSCs are thought to play a central role in the resistance to conventional therapies, underscoring the need for targeted treatments that can specifically eradicate this resistant population of cells.

Among the various cell-based assays, the ChemoID assay has gained particular attention for its ability to assess drug sensitivity in GSCs [10]. This assay evaluates fresh tumor biopsies from patients against a panel of FDA-approved chemotherapies, allowing clinicians to identify which drugs are most effective in targeting the cancer stem cells within the tumor. By focusing specifically on CSCs, the ChemoID assay provides a means of tailoring treatments to the individual patient’s tumor biology, potentially improving therapeutic outcomes, particularly in the setting of recurrent GBM [11–15]. Several studies have demonstrated that the ChemoID assay can offer valuable insights into tumor behavior, correlating drug sensitivity profiles with clinical outcomes and providing a promising strategy for personalizing therapy in the fight against GBM.

While these advances in cell-based assays hold great promise, challenges remain in terms of standardizing methodologies and ensuring the reproducibility of results across different research settings. Furthermore, while the use of targeted therapies guided by these assays could improve the management of GBM, further validation and clinical trials are needed to establish their widespread use in routine clinical practice. Nevertheless, the increasing focus on personalized treatment strategies based on cell-based drug screening is a crucial step forward in overcoming the significant hurdles posed by the molecular complexity and resistance mechanisms of brain tumors [16–18].

This systematic review aims to gather the current evidence on the use of cell-based assays for targeted therapies in central nervous system tumors, with a focus on their ability to predict treatment response in recurrent GBM. We explore how these assays can help personalize treatment regimens, ultimately improving clinical outcomes for patients with recurrent GBM.

## Results

**Fig. 1:**
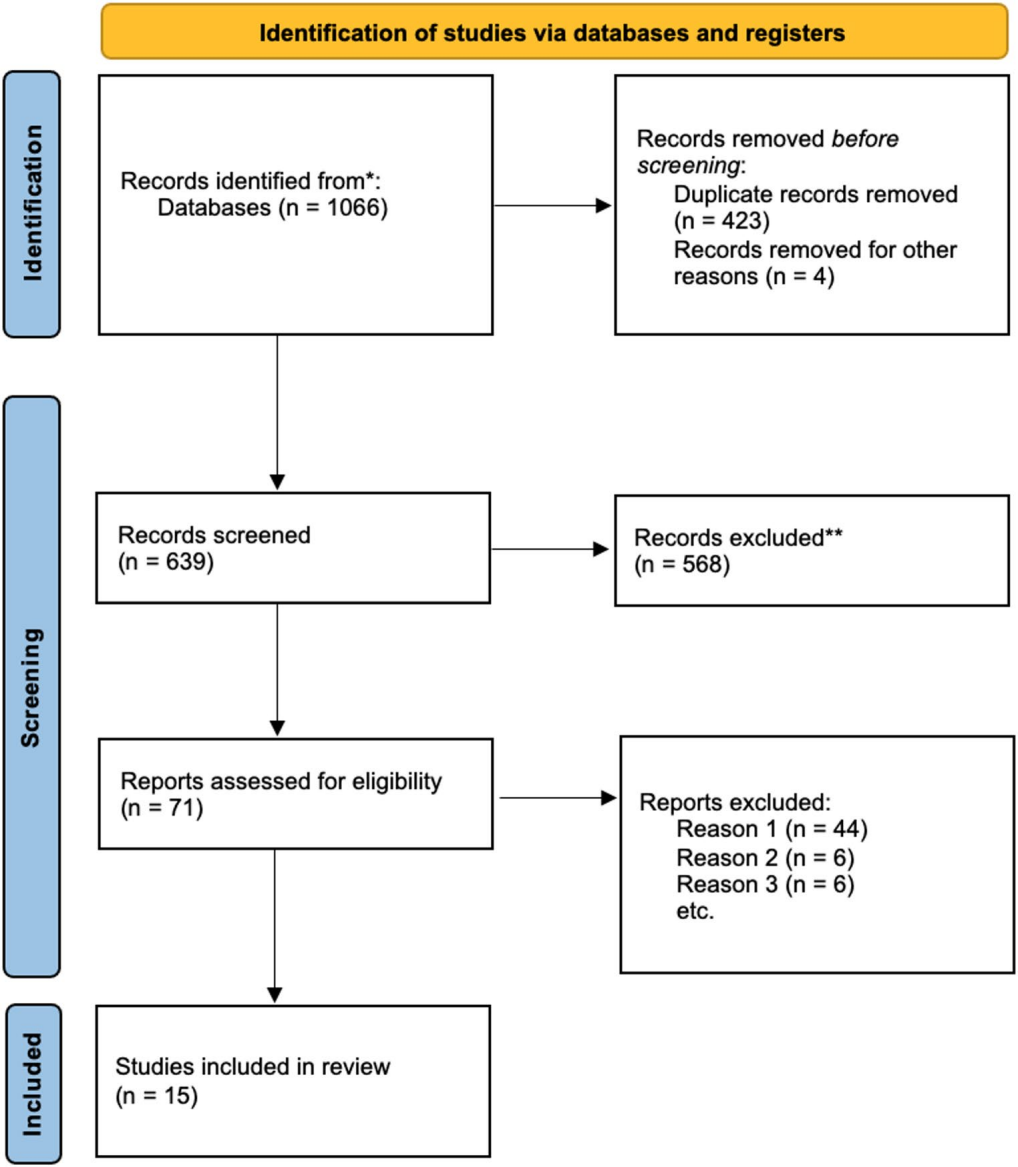
PRISMA 2020 flow diagram. Reason 1: non-cell-based therapies, Reason 2: not reported patient clinical data, Reason 3: not reported patient outcomes

The comprehensive search yielded 1066 articles. After removal of duplicate and other ineligible products (*n* = 427), 639 abstracts were screened, and 71 full-text articles were identified for review. Of these, 56 were removed for the following reasons: 44 articles concerned non–cell-based therapies (Reason 1), 6 articles did not report patients’ clinical data (Reason 2), and 6 did not report patients’ outcomes (Reason 3). Ultimately, this resulted in the inclusion of 15 studies for detailed analysis (Fig. 1), comprising different study designs including case reports, cohort studies, prospective clinical trials, and a single randomized controlled trial. These studies (Table 1) collectively involved 300 patients, with sample sizes ranging from 1 to 78 patients. The studies focused on a variety of tumor types, including rGBM, diffuse astrocytoma progressing to high grade, ependymoma, and anplastic chordoma, although the majority concentrated on rGBM. Various cellular models were employed, including patient-derived organoids, neurosphere-forming cultures, GSCs, to test ex vivo sensitivity to therapies.

**Table 1:** Studies included in the systematic review

First Author and Date	*N*. Patients	Median Age	Histology	Cellular Model	Model of the study	Therapy tested	Results
Peng et al., 2025 [19]	10	NA	Brain tumors (oligodendroglioma, pilocytic astrocytoma, ependymoma, medulloblastoma, GBM)	Organoids IPTOs	Prospective clinical study	Several drugs	IPTOs could predict patient-specific responses to therapies such as temozolomide and radiotherapy, targeted agents (as Osimertinib).
Toh et al., 2024 [20]	10	NA	High Grade Astrocytic Glioma (HGG)	Organoids	Interventional, non-randomized, open-label trial	Several drugs	Study protocol for GBOs-guided chemotherapy using both standard and experimental drug panels
Logunet al., 2024 [21]	6	NA	rGBM	Organoids	single-arm, phase I clinical trial, dual-targeting CAR-T cells (EGFR-IL13Ra2)	dual-targeting CAR-T cells (EGFR-IL13Ra2)	GBOs can serve as real-time avatars for assessing CAR-T therapy efficacy (demostrated with significant cytolysis).
Ranjan et al., 2023 [11]	78	57.5	rGBM	ChemoID assay	randomized clinical trial, the ChemoID assay vs. over physician-chosen chemotherapy	Several drugs	ChemoID use was related to increased patient OS compared to control
Mathews et al., 2023 [12]	1	25	rGBM	ChemoID assay	case report, ChemoID assay-guided chemotherapy regimen	Lomustine, Procarbazine	ChemoID use was related to increased patient OS compared to control (lomustine and procarbazine vs. TMZ treatment).
Ntafoulis et al., 2023 [22]	55 (66 assays)	58	rGBM	Patient-derived GSCs (low-passage)	Prospective observational clinical study	Temozolomide	Ex vivo TMZ sensitivity (IC50, AUC, viability) significantly correlated with OS and PFS. Identified 3 predictive groups (responders, intermediate, non-responders). Predictive performance superior to MGMT methylation.
Liu et al. 2022 [23]	8	49,8	rGBM	Primary GBM cultures	single-arm, open-level phase I clinical trial	TMZ, BCNU	IC50 of drugs correlated with chemotherapy resistance.
Shuford et al., 2021 [24]	20 (33 assays)	56	High-grade glioma/GBM	Ex vivo 3D patient-derived cultures	Prospective clinical observational study (NCT03561207)	Temozolomide and other agents	Ex vivo assay predicted clinical response with 85% accuracy. Test-predicted responders showed significantly longer OS (11.6 vs. 5.9 months). Provided actionable profiles also in recurrent cases.
Zhang et al., 2021 [25]	1	n.d	GBM	Patient-derived GBM cells into cerebral organoids	case report	TMZ	therapy tested on GBM cell- transplanted cerebral organoids impact on patient outcomes
Ranjan et al. 2020 [13]	14	49	12 GBM and 2 progressive anaplastic glioma	ChemoID assay	case report	9 single drugs and and 5 drug combinations	ChemoID use was related to increased patient OS compared to control.
Tsoli et al. 2018 [26]	1	10	rGBM	Neurosphere-forming GBM culture	case report	128 anti-cancer drugs	12 drugs were found to be more effective, unclear clinical correlation.
D’Alessandris et al. 2017 [27]	52	59.5	rGBM	GSCs	Cohort study	Radiation and TMZ	GSCs with lower IC50 to TMZ and LD50 to radiation were associated with longer patient survival
Howard et al. 2017 [14]	41	54	GBM	ChemoID assay	Cohort study	TMZ	ChemoID TMZ-sensitive patients had a significantly improved prognosis than ChemoID TMZ-resistant patients after treatment with TMZ.
Mathis et al. 2014 [15]	2	10,7	anaplastic WHO grade 3 ependymoma	ChemoID assay	case report	saline solution, oxaliplatin; bevacizumab; irinotecan; cisplatin, combination	1st patient ChemoID use was related to increased patient OS compared to control. 2nd patient not sensitive to any of the chemotherapies tested and rapidly progressed
Ricci-Vitiani et al. 2013 [28]	1	30	chordoma	chordoma cell line obtained during first surgery	case report	Rapamycin and other drugs	Rapamycin was identified as effective on cell line and showed also clinical efficacy

The reported treatments encompassed standard chemotherapeutics (e.g., temozolomide, lomustine, procarbazine), targeted agents (e.g., osimertinib, gefitinib, rapamycin), and combination regimens. While some studies described post-hoc correlations between GSC sensitivity and patient outcomes, most investigated prospective GSC- or PDO-guided treatments. Notably, the ChemoID assay, a partially standardized GSC-based test, emerged as the most frequently applied platform across multiple studies and was associated with improved patient survival when guiding therapy.

Despite the heterogeneity in models and methods, a consistent and non-negligible correlation was observed between ex vivo cellular sensitivity and clinical response. The overall quality of evidence remains limited, with nearly half of the studies being case reports.

## Discussion

Recent advances in the understanding of molecular biology of tumors have allowed to identify driver mutations in a number of central nervous system tumors; instead, no driver mutation has been found in GBM [29]. Accordingly, molecular patients stratification, which has been extensively adopted in GBM trials in recent years, has not led to a substantial improvement of GBM prognosis so far [30, 31]. Moreover, retrospective molecular analysis of clinical trials failed to identify robust predictive factors for response to targeted drugs [32, 33]. To overcome these drawbacks, cell-based assays to assess tumor drug sensitivity have been recently proposed [34, 35]. From a theoretical viewpoint, cell-based assays could provide substantial advantages. Reportedly, besides interpatient heterogeneity, GBM holds a remarkable intratumoral heterogeneity. The small fraction of GBM cells with stem-like features, the GSCs, is highly resistant to radiotherapy and chemotherapy [9, 36] and thus it is responsible for tumor recurrence after treatments. Therefore, in principle, a treatment active against GSC should target the actual cell population which gives birth to tumor recurrence, and thus should be highly effective. In summary GSCs, besides representing the ideal model for preclinical GBM investigations, have the potential to give key information on drug sensitivity of the tumor of origin, particularly in the setting of recurrence, which is enriched of cells with stemlike features.

Our comprehensive systematic literature review found 15 papers describing some interaction between patient and cell drug sensitivity. The overall quality of evidence has to be considered poor, since most papers are case reports. Moreover, no agreement on a standard method to define the chemosensitivity of cell cultures, nor on the type of cells to be used (GSC vs. primary cultures) exist.

The ChemoID assay, which was adopted in five of the reviewed studies, is a clinically validated method that specifically identifies cytotoxic therapies targeting cancer stem cells (CSCs) within solid tumors, by evaluating fresh cancer biopsies against a panel of FDA-approved chemotherapies. Several studies have evaluated this method in a number of solid tumors, including brain tumors. In a randomized clinical trial (NCT03632135), Ranjan et al. [11] found that the ChemoID assay-guided group had improved survival compared to the physician-choice group. The median survival was 12.5 months versus 9 months, respectively, and the ChemoID-guided group had a significantly lower risk of death. In a 2020 case series [13], the same group used ChemoID to guide chemotherapy choices in GBM and progressive anaplastic glioma patients, resulting in improved overall survival compared to historical data. Mathews et al. [12] applied ChemoID to treat a patient with recurrent high-grade glioma, guiding targeted treatment with lomustine and procarbazine. The ChemoID use was related to increased patient survival compared to control (TMZ). Howard et al. [14] conducted a prospective study in GBM patients treated with standard-of-care TMZ plus radiation. ChemoID CSC test results correlated with progression-free survival (PFS), and overall survival (OS). In particular, ChemoID TMZ-sensitive patients had a significantly improved prognosis than ChemoID TMZ-resistant patients after treatment with TMZ. Finally, Mathis et al. [15] applied ChemoID to two patients with anaplastic WHO grade 3 ependymoma. The use of ChemoID in the first patient was related to an increase in patient OS compared to control. The second patient was not sensitive to any of the chemotherapies tested and had rapid progression of the disease.

In the other reviewed studies, the techniques for analyzing the sensitivity of cells to chemotherapeutics were different. Logun et al. [21] describe a unique phase 1 clinical trial in which tumor samples from six patients with recurrent glioblastoma were collected during surgery and used to rapidly generate patient-derived glioblastoma organoids (GBOs). These organoids were treated ex vivo with the same dual-targeting CAR-T cells (EGFR-IL13Ra2) administered to the patients. The study found that CAR-T cell treatment led to significant cytolysis and reduction of target antigens in the organoids, with these effects strongly correlating to CAR-T cell engraftment and clinical responses in patients. The results demonstrate that GBOs can serve as real-time avatars for assessing CAR-T therapy efficacy. Peng et al. (2025) [19] developed individualized patient tumor organoids (IPTOs) from brain tumor samples, including glioblastoma and other types. These organoids faithfully preserved the cellular heterogeneity and tumor microenvironment of the original patient tumors. The study demonstrated that IPTOs could predict patient-specific responses to therapies such as temozolomide and radiotherapy, targeted agents (as Osimertinib). In a prospective clinical cohort, patients whose IPTOs responded to treatment showed significantly improved overall survival and progression-free survival. This innovative model offers a promising platform for personalized therapy testing and precision medicine in neuro-oncology, enabling more accurate prediction of clinical outcomes and better treatment selection. Another study that utilized organoids was conducted by Toh et al. [20], in which patient-derived organoids were tested with two distinct drug panels. The first panel consisted of chemotherapeutic agents commonly used for glioblastoma (GBM), including SN-38, lomustine, temozolomide, vincristine, procarbazine, etoposide, carboplatin, cisplatin, cyclophosphamide, docetaxel, capecitabine, and vinorelbine. The second, termed the “experimental panel,” comprised anti-cancer agents with promising activity in high-grade glioma (HGG) that are not yet routinely used in clinical practice. These included gefitinib, osimertinib, vemurafenib, everolimus, sunitinib, regorafenib, selinexor, marizomib, abemaciclib, ivosidenib, olaparib, and metformin. However, only the study protocol is reported, and no results of this study are currently available.

Liu et al. [23] investigated the treatment of recurrent GBM using primary GBM cultures. Their single-arm, open-label phase I clinical trial evaluated the efficacy of temozolomide (TMZ) and carmustine (BCNU). Notably, the IC50 of these drugs correlated with chemotherapy resistance.

Zhang et al. [25] reported a real-time integrated system by generating 3D ex vivo cerebral organoids and in vivo xenograft tumors based on glioma patient-derived tissues and cells. They identified a case from a grade 2 astrocytoma patient with typical grade 4 GBM features in both organoids and xenograft models, which mimicked the disease progression of this patient. Similarly, Shuford et al. (2021) prospectively showed that a 3D patient-derived culture assay could predict temozolomide responsiveness with 85% accuracy. Predicted responders had nearly double the median overall survival, and the assay proved informative even in patients with recurrent disease.

Tsoli and colleagues [26] investigated the treatment of recurrent GBM using neurosphere-forming GBM cultures. They evaluated the efficacy of 128 anti-cancer drugs, with 12 of them showing greater effectiveness, although the clinical correlation remains unclear.

Our research group has exploited the cancer stem cell model using a cell viability test based on a high-throughput bioluminescence assay in GBM, plus an in vivo model in chordoma. The GBM study [27] investigated 52 patients treated with surgical resection followed by standard-of-care radiotherapy and temozolomide (TMZ), from which a GSC culture could be established. Notably, we found an inverse correlation between GSC IC50 to TMZ and suvival. Moreover, we were able to find cut-off values for IC50 to TMZ and LD50 (50% lethal dose) to radiotherapy, able to identify patients likely to respond to chemoradiation. Importantly, cases with TMZ half-maximal inhibitory concentration < 50 µM (within the range of plasma levels achieved in vivo) had longer OS and PFS. The chordoma study [28] was a case report focused on a patient harboring recurrent clivus chordoma after multiple surgeries and proton-beam radiotherapy. Since no clinically validated chemotherapy regimens for chordoma are available, we challenged chordoma stem-like cells with a library of small kinase inhibitors. This assay identified rapamycin as the most active drug; the effectiveness of the drug was validated in an in vivo subcutaneous chordoma xenograft. Therefore, the patient received rapamycin therapy with about six-fold reduction of the tumor growth rate upon 10-month follow-up neuroimaging.

Ntafoulis et al. [22] demonstrated in a cohort of 55 patients that temozolomide sensitivity measured ex vivo on low-passage GSC cultures strongly correlated with overall and progression-free survival, outperforming MGMT promoter methylation as a predictive biomarker and enabling a clinically meaningful stratification into responders, intermediate, and non-responders.

Of note, in 2025 a review by Bardhan et al. [37] was published on the use of stem cell-based therapies for GBM. The review highlights how various stem cell types—neural, mesenchymal, hematopoietic, and adipose-derived—are being explored for their ability to target GSCs, which contribute to tumor resistance and recurrence. These therapies work through mechanisms such as tumor targeting, immune modulation, angiogenesis inhibition, and apoptosis induction. Despite promising early results in clinical trials, challenges remain, including safety concerns and delivery methods. The focus of this review, instead, is not the use of stem cell itself as therapeutic agents, but the use of stem-cell based models for therapy personalization in neuro-oncology.

As limitations of the present study, we must disclose the overall quality of results from the systematic review of the literature was low.

This being the state-of-the-art, much work remains to be done to put cell-based assay at neuro-oncologists disposal during routine clinical work in their fight against brain tumors.

In conclusion, though still preliminary, the available evidence highlights the potential of cell-based drug screening assays as a promising approach to personalize therapy for brain tumors—particularly for GBM. Unlike traditional molecular profiling, which often fails to capture the complexity and therapeutic vulnerabilities of these tumors, especially at recurrence, in vitro assays using patient-derived models such as GSCs and organoids can provide functional insights into treatment sensitivity. This is especially valuable in GBM, where intratumoral heterogeneity and lack of clear molecular targets limit the effectiveness of current therapies. Although the overall quality of published studies remains limited and further standardization is needed, this strategy represents a significant step forward in tailoring treatments to individual patients. With continued clinical validation, cell-based assays could become a valuable tool in the neuro-oncology setting, supporting more precise and effective therapeutic decision-making for brain tumors as a whole, and GBM in particular.

## Methods

This systematic review was conducted according to PRISMA guidelines to evaluate whether functional, cell-based drug-sensitivity assays can inform personalized therapeutic strategies for glioma patients. A comprehensive search was performed across two major biomedical databases—PubMed and Scopus—to ensure maximal coverage and minimize database-specific bias. Search terms included combinations of (target/targeted therapy/drug/treatment OR personalized therapy/treatment) AND (cell-based/cell line/stem cell OR in vitro OR ex vivo OR xenograft OR PDX) AND (glioma OR glioblastoma OR brain tumor) AND (clinical study OR patient OR trial). The review protocol has been submitted in PROSPERO database (https://www.crd.york.ac.uk/prospero, registration no. 573177).

Studies were eligible if they: (1) included a functional, cell-based drug-response assay performed on patient-derived material; (2) reported original clinical data; and (3) provided a correlation between assay results and patient-level outcomes (OS, PFS, or radiological response). Exclusion criteria included purely preclinical studies, lack of patient outcome data, non–cell-based assays, review articles, and non-English publications.

To ensure methodological rigor, two independent reviewers (M.O., S.S.) performed study selection in two stages (title/abstract screening and full-text assessment). Discrepancies were resolved by consensus, and when agreement was not reached, a third senior reviewer (Q.G.D.) adjudicated. In addition, each included study was qualitatively evaluated for methodological consistency, completeness of outcome reporting, and potential sources of bias (e.g., sample size, prospective vs. retrospective design, assay reproducibility), although the heterogeneity of study designs precluded the use of a single standardized risk-of-bias tool.

The search, completed in June 2025, yielded 1066 records. After removal of duplicates and ineligible entries, 639 records were screened and 71 full texts were assessed for eligibility. Of these, 56 were excluded based on predefined reasons, leaving 15 studies for qualitative synthesis.

## Data Availability

No data were generated for this study.
